# The *PTPN22* C1858T (R620W) functional polymorphism in inflammatory bowel disease

**DOI:** 10.1186/s13104-018-3875-7

**Published:** 2018-11-01

**Authors:** Younes Zaid, Nezha Senhaji, Fatima Zahra Bakhtaoui, Aurora Serrano, Nadia Serbati, Mehdi Karkouri, Wafaa Badre, Mounia Oudghiri, Javier Martin, Sellama Nadifi

**Affiliations:** 1 Research Center of Abulcasis University of Health Sciences, Rabat, Morocco; 2 Laboratory of Genetic and Molecular Pathology (LGPM), Faculty of Medicine and Pharmacy of Casablanca, Hassan II University, Casablanca, Morocco; 3 Anatomical Pathology Laboratory, CHU Ibn Rochd, Casablanca, Morocco; 4 Instituto de Parasitología y Biomedicina López-Neyra, Consejo Superior de Investigaciones Científicas, P.T.S. Granada, Granada, Spain; 5 Gastroenterology Department, CHU IbnRochd, Casablanca, Morocco; 6 Department of Biology, Immunology and Biodiversity Laboratory, Faculty of Sciences, Hassan II University, Casablanca, Morocco

**Keywords:** *PTPN22*, IBD, CD, UC, Morocco

## Abstract

**Objective:**

In view of the discrepant data regarding the association between the protein tyrosine phosphatase non-receptor 22 (*PTPN22*) rs2476601 (R620W, 1858C→T) polymorphism and susceptibility to autoimmune diseases including inflammatory bowel diseases (IBD), we investigated whether this functional single-nucleotide polymorphism influences IBD risk in a group of Moroccan patients.

**Results:**

This is the first report on the prevalence of *PTPN22* (R620W) variant in a Moroccan cohort. No evidence of statistically significant differences was observed when the *PTPN22* (R620W) allele and genotype distribution among IBD, Crohn’s disease (CD), ulcerative colitis (UC) patients and healthy controls were compared. The frequency of the variant allele in healthy subjects was 1.77% compared to 2.56% in the IBD patients and 1.85% in CD patients. Furthermore, the frequency of this allele was increased in UC patients compared to controls (4.17% vs. 1.77%, OR = 2.42, 95% CI 0.82–7.08; P = 0.09), but the difference was not statistically significant. Our data suggest a lack of association between *PTPN22* R620W variant and IBD susceptibility in Moroccan patients.

## Introduction

The inflammatory bowel diseases (IBD) are chronic inflammatory disorders of the gastrointestinal tract that manifests as Crohn’s disease (CD) and Ulcerative colitis (UC), clinically characterized by periods of remission punctuated by episodes of clinical disease activity [1]. IBD are likely thought to result from interplay of several immunological, environmental, genetic, and life-style factors that may proceed to a dysregulated mucosal immune response to the gut microflora [2].

Dissimilarities are observed between CD and UC in terms of disease manifestation. This Heterogeneity may reflect differences in the pathogenesis between the two forms of IBD. There is some support for the concept that differences between CD and UC may be in large measure genetically determined [3].

As in most autoimmune diseases, genetic susceptibility to IBD is heterogeneous and complex and presumably implies multiple genes of relatively low penetrance [4]. Genome-wide scans and linkage analyses have identified a number of genetic markers as possible IBD susceptibility loci, with some of them observed uniquely in UC or CD, and others common to both disorders [5].

Analysis of selected candidate genes mapping within susceptibility regions revealed variant in the *PTPN22* (protein tyrosine phosphatase non-receptor 22) gene as being associated with IBD [6]. Located on chromosome 1p13, *PTPN22* encodes an intracellular protein tyrosine phosphatase (PTP) expressed in T lymphocytes, the Lymphoid-tyrosine phosphatase (LYP) with a molecular weight of 110 kDa [7], which is implicated in maintaining the resting phenotype of lymphocytes [8].

The intracellular PTP, LYP, contains a catalytic N-terminal and non-catalytic C terminus domains with four proline-rich motifs [9]. LYP is found to be physically bound with high affinity to the SH3 domain of the Src kinase (CSK) through one prolinerich motif (referred to as P1). These interactions regulate the phosphorylation state of the kinases LCK, Fyn and Zap-70, all known to be important in T cell receptor signaling [10].

Protein tyrosine phosphatase are critical regulators of many different aspects of T-cell physiology. In conjunction with protein tyrosine kinases (PTKs), PTPs regulate the reversible phosphorylation of tyrosine residues in proteins and thereby play important roles in T-cell signal transduction [11].

Abnormalities in tyrosine phosphorylation have been demonstrated to be involved in the etiological and pathogenic process of a range of diseases in which the phenotypic spectrum includes an aberrant immune response. Thereby, T cells displaying dysregulated tyrosine phosphorylation, and hence abnormal T cell activation, would predictably mediate the pathological process in several autoimmune diseases.

Several lines of evidence suggest that the functionally relevant *PTPN22* (R620W) polymorphism may lower the threshold of T-cell activation and hence may heighten an individual carrier’s risk for human autoimmune and infectious disorders [11].

A missense single nucleotide polymorphism (SNP) with functional influence, the R620W (rs2476601, 1858C>T) in exon 14 of *PTPN22* gene, changes the amino acid at position 620 from an arginine (R) to a tryptophan (W). The latter variant disrupts the interaction between LYP and CSK, which prevents formation of the complex and, therefore, suppression of T-cell activation [12]. In vitro experiments have shown that 620W-allele binds less efficiently to CSK than R-allele suggesting that T cells expressing the risk allele may be hyperresponsive [13]. Consequently, individuals carrying this allele may be prone to multiple autoimmune disorders.

Functional studies of mutant Lyp in T cells showed that the *PTPN22* T1858 acts in an autosomal dominant fashion and confers significant predisposition to autoimmunity even when present as a single copy [14] with increased clinical penetrance in homozygous carriers [15].

Investigation of *PTPN22* R620W (C1856T) association with CD was first reported by van *Oene M* et al. [16] subsequent studies have had divergent results and showed strong evidence of ethnic differences. An interesting feature of the *PTPN22* R620W (C1856T) polymorphism is the wide variation of allele frequency among different populations, i.e. there is a gradient of increasing frequency in white European populations. In most American–European populations, allele frequencies range between 8 and 9%. In addition, the *PTPN22* 620W allele was reported to be absent from Asian and African populations [13, 12]. These large allele frequency differences in the various populations emphasize the importance of careful case–control matching for genetic studies, particularly in the North African population where considerable population heterogeneity has been reported [17]. Accordingly, In view of the discrepant data regarding the distribution of the risk alleles of *PTPN22* R620W we tested for the first time the association of the polymorphism with the susceptibility to IBD in a cohort of Moroccan patients in order to determine quantitatively the risk of CD and UC with the rs2476601 variant under an allelic, recessive and dominant model.

## Main text

### Results

We examined the possibility that the *PTPN22* 1858T variant might predispose to IBD, by genotyping 135 patients with CD and 60 subjects with UC and 311 controls with Moroccan origin. Genotype assignment was successful in > 95% of samples tested.

The sample size used in the IBD analyses had 50% power to detect the effect of the SNP conferring an odd ratio 1.7 at the 5% significance level.

Allele and genotype distributions for the *PTPN22* polymorphism in cases and controls are summarized in Tables 1 and 2. Overall, we observed no evidence of genetic association between the *PTPN22*1858T polymorphism and IBD susceptibility. When comparing patients with the control group, frequencies of the *PTPN22* risk allele were not significantly different: the frequency of the variant 1858T allele amounted to 1.85% in CD cases, 2.56% in the combined IBD, and 1.77% in healthy controls. Noteworthy, the frequency of the *PTPN22* 620-W allele tended to be higher in UC cases than in controls, without reaching a significant difference (4.17% vs. 1.77%, OR: 2.42, 95% CI 0.82–7.08; P = 0.09).

**Table 1:** Minor allele frequencies of *PTPN22* (rs2476601) genetic variant in IBD patients and healthy controls from Morocco

*SNP ID*	Subgroup	Number of alleles	MAF %	Allele test	
				OR [95% CI]	P-value
*PTPN22* rs2476601	Controls (n = 311)	11/611	1.77		
	IBD (n = 195)	10/380	2.56	1.46 [0.61–3.48]	0.38
	CD (n = 135)	5/265	1.85	1.05 [0.36–3.05]	0.93
	UC (n = 60)	5/115	4.17	2.42 [0.82–7.08]	*0.09*

Frequencies of the *PTPN22* allele between CD patients, UC patients, and IBD patients, were not significantly different compared to healthy controls

Italic value indicates trend of association (P = 0.09)

*SNP ID* Single nucleotide polymorphism identifiant, *IBD* inflammatory bowel disease, *CD* Crohn’s disease, *UC* ulcerative colitis, *OR* odds ratios, *PTPN22* protein tyrosine phosphatase non-receptor type 22, *MAF* Minor Allele Frequencies

**Table 2:** Genetic models and genotypes distribution in patients and controls

Gene SNP	Group	1/2	Genotype, N (%)			Dominant model Genotype 11 + 12 vs. 22		Recessive model Genotype 11 vs. 12 + 22	
			AA	AG	GG	OR (95% CI)	P-value	OR (95% CI)	P-value
*PTPN22* rs2476601	Controls	A/G	1 (0.32)	9 (2.89)	301 (96.78)				
	IBD		1 (0.51)	8 (4.10)	186 (95.38)	1.45 [0.58–3.65]	0.42	1.59 [0.09–25.7]	0.74
	CD		0 (0.00)	5 (3.70)	130 (96.30)	1.15 [0.38–3.45]	0.79	NA	NA
	UC		1 (1.69)	3 (5.08)	56 (94.91)	2.15 [0.65–7.09]	0.20	5.25 [0.32–85.18]	0.24

No association was seen with *PTPN22* SNP under dominant model AA + AG vs. GG (11 + 12 vs. 22) or recessive AA vs. AG + GG (11 vs. 12 + 22) either for the IBD group or when dividing into CD and UC groups

*PTPN22* protein tyrosine phosphatase non-receptor type 22, *SNP* Single nucleotide polymorphism, *IBD* inflammatory bowel disease, *CD* Crohn’s disease, *UC* ulcerative colitis, *OR* odds ratios

Similarly, no statistically significant differences were observed when the 1858C>T SNP genotype distribution between CD patients, UC patients, and healthy controls was compared. Furthermore, combined phenotype of CD and UC (IBD patients) displayed no significant difference in genotype frequencies of *PTPN22* 1858C>T polymorphism compared with controls. Worth mentioning that, the homozygous genotype for the variant allele was absent in CD patients.

Regarding the possible effects of genetic models on disease risk, no association was seen with *PTPN22* SNP under recessive AA vs. AG + GG or dominant model AA + AG vs. GG, either for the IBD group as a whole or when dividing into CD and UC groups.

### Discussion

The *PTPN22* (R620W) SNP was first reported to be associated with type 1 diabetes [18] and later with rheumatoid arthritis [13], systemic lupus erythematosus [19], autoimmune thyroid disease [20] and as such with several other autoimmune diseases [21]. However, the mechanism by which altered 620W*PTPN22* function in lymphocytes might contribute to the diverse *PTPN22*-associated pathologies remains not fully determinate. On the other hand, the genetic implication of this variant could not be evidenced in some inflammatory diseases such as psoriasis, multiple sclerosis and Behcet’s disease [22, 23]. These differences may be the result of different adaptive immune system pathogeneses, in particular regarding T cells, in these disorders.

Given these data, *PTPN22* (R620W) polymorphism can be considered as good candidate gene in the study of genetically determined IBD. In view of the etiologic and pathogenic relevance of *PTPN22*, we sought to assess the potential role of the 620W as a candidate susceptibility allele for IBD, CD and UC in a case–control study of Moroccan patients.

Our study revealed no direct association of the *PTPN22* (R620W) polymorphism with IBD in our patient population (see Table 1). In addition, no differences were observed in the distribution of genotypes between patients and healthy controls. Interestingly, our findings are in agreement with studies on Spanish [21, 24] British [25] Northern German [26] Canadian [27] New Zealand [28] and Czech [29] populations, wherein this association was not registered.

This was also confirmed by the meta-analysis conducted by Latiano et al., showing a lack of association between the *PTPN22* C1858T variant and the CD and UC phenotypes [30].

It should be noted that the absence of the homozygous genotype for the variant allele in our CD cohort is in accordance with its absence in Asian and African populations, with the polymorphic allele being most prevalent in Scandinavia [31].

On the other hand, our results are in disagreement with previous reports where it has been shown that the polymorphism of the PTPN22 (R620W) gene rather played a predisposing factor to IBD risk.

Sfar et al., argued for the association between *PTPN22* 1858T allele and susceptibility to IBD in Tunisian patients [32]. However, the absence of homozygous genotype for the variant allele is also found in this latter North African population. *PTPN22 SNP* has also been associated with colonic CD [33] in a Canadian cohort.

Otherwise, a genetically determined lower activity of the inflammatory gene *PTPN22* (620-W) was associated with reduced risk of CD and UC in a Danish Cohort [34]. In a similar way, Meta-analysis testing the overall effect of the *PTPN22* 620W variant pointed out an association with reduced risk of CD but not of UC [35].

To date it is thoroughly known that the frequency of the examined PTPN22 (R620W) variant varies between populations. Contradictory results have been published regarding the association of rs2476601 variant with either CD or UC. This inconsistency might be due to differences in sample size, selection bias or allele frequencies. Overall, there is inescapable evidence for the considerable effect of population stratification on case–control association studies.

Our study showed that frequency of the mutated allele is higher within IBD and particularly in UC patients compared to controls. However, although higher frequencies of the variant 1858T allele were noticed, no significant difference in allelic distribution was identified in our case–control study.

The discrepancy between our findings and previous studies might be explained not only by the difference in minor allele distribution of *PTPN22* rs2476601 in our population but also by the contribution of genetic factors in disease-related ethnic disparities. Those data further show the real difficulties encountered in analyzing candidate genes in complex diseases. Thereby, further well powered studies are needed to obtain a clear insight into the impact of the Protein Tyrosine Phosphatase Non-receptor type 22 (PTPN22) on the immunogenetic and pathophysiological aspects of IBD.

### Materials and methods

#### Subjects

Our study population consisted of 195 IBD [135 CD and 60 UC] patients recruited from the CHU Ibn Rochd Hospital (Casablanca, Morocco). The ethnically matched control group was described before [36] and comprises 311 unrelated healthy subjects with Moroccan origin. None of the control individuals had any evidence of autoimmune diseases.

Diagnosis of CD or UC was confirmed in accordance with the standardized set of clinical, radiological, endoscopic, and histological criteria [37]. Data obtained from each patient were collected in a case report form as previously described [38] and included age at diagnosis, disease location and phenotype, extra-intestinal manifestations, toxic behavior and other clinical features.

#### Ethics statement

All study participants provided written informed consent. The study was approved by the Ethics Committee of the Faculty of Medicine and Pharmacy of Casablanca and was conducted in accordance with the Declaration of Helsinki.

#### Genotyping

Genomic DNA samples were obtained from peripheral whole-blood using the salting-out method and from Formalin Fixed Paraffin Embedded Tissues using the QIAamp DNA FFPE Tissue Kit (Qiagen, Valencia, CA, USA). DNA concentration and quality were analyzed using a NanoDrop 1000 spectrophotometer (Thermo Fisher Scientific, Wilmington, DE, USA).

Genotyping of the *PTPN22* SNP (rs2476601, 1858C→T, R620W) was performed using the TaqMan 5′-allele discrimination (predesigned TaqMan™ SNP Genotyping Assay by Applied Biosystems Catalog Number: 4351379) on the Light Cycler 480 System (Roche, Barcelona, Spain). The polymerase chain reaction (PCR) was carried out in a total reaction volume of 5 μl with 0.01 μg/μl of genomic DNA, using the following cycling conditions: one cycle of 95 °C for 3 min followed by 50 cycles of 95 °C for 3 s, 60 °C for 20 s, and cooled down to 4 °C for storage. To detect sequence variations the LightCycler^®^ 480 software release 1.5.0 SP4 version 1.5.0.39 was used.

#### Statistical analysis

Patients were grouped into CD, UC and all IBD subjects to determine differences in the minor allele frequencies between the different groups. Hardy–Weinberg equilibrium (HWE) assessment was done using the Pearson’s Chi square test. Statistical power was calculated using Power Calculator of Genetic Studies 2006 software (http://www.sph.umich.edu/csg/abecasis/CaTS/). Allele frequency and genotype distribution were estimated for the disease compared to the control group based on χ^2^ analysis or Fisher’s test. Descriptive statistical analysis has been performed using Plink software V1.07 (http://pngu.mgh.harvard.edu/purcell/plink/). Association analyses were applied to detect the associations with the candidate SNP and disease risk. The strength of association was estimated by calculation of the Relative risk/odds ratios (OR) and 95% confidence intervals (CI). A P-value < 0.05 was considered to be statistically significant.

## Limitations

Although the capacity of the *PTPN22* (R620W) variant to impair Lyp function strongly supports its relevance in the etiology of a subgroup of autoimmune diseases, the pathogenic inflammatory pathway is not always common between these disorders. Based on the evidence of this study and former reports, it is likely that the functional effect of this polymorphism is not a major contributing factor to IBD susceptibility in the Moroccan population.

In view of the possible confounders’ effect on the present study, it is likely that due to small sample size, an association cannot be ruled out. Further well-powered studies would be required to confirm our findings.

## Abbreviations

IBD: inflammatory bowel disease
CD: Crohn’s disease
UC: ulcerative colitis
OR: odds ratios
PTPN22: protein tyrosine phosphatase non-receptor type 22
PTP: protein tyrosine phosphatase
LYP: lymphoid-tyrosine phosphatase
SNP: single nucleotide polymorphism
